# Looking after bubba for all our mob: Aboriginal and Torres Strait Islander community experiences and perceptions of stillbirth

**DOI:** 10.3389/fpubh.2024.1385125

**Published:** 2024-04-16

**Authors:** Luciana Massi, Carolyn Lewis, Skye Stewart, Diana Jans, Rupesh Gautam, Lina Jalloub, Anneka Bowman, Philippa Middleton, Sue Vlack, Frances M. Boyle, Carrington Shepherd, Vicki Flenady, Deanna Stuart-Butler, Kym M. Rae

**Affiliations:** ^1^Stillbirth Centre of Research Excellence, Mater Research Institute, The University of Queensland, Brisbane, QLD, Australia; ^2^Indigenous Health Research Group, Mater Research Institute, The University of Queensland, Brisbane, QLD, Australia; ^3^Curtin Medical School, Curtin University, Perth, WA, Australia; ^4^Red Nose Australia, Melbourne, VIC, Australia; ^5^Apunipima Cape York Health Council, Cairns, QLD, Australia; ^6^Department Aboriginal Communities and Families Research Alliance, South Australia Health and Medical Research Institute, Adelaide, SA, Australia; ^7^Pregnancy and Perinatal Care, South Australia Health and Medical Research Institute, Adelaide, SA, Australia; ^8^School of Public Health, The University of Queensland, Brisbane, QLD, Australia; ^9^Institute for Social Science Research (ISSR), The University of Queensland, Brisbane, QLD, Australia; ^10^Ngangk Yira Research Institute, Murdoch University, Perth, WA, Australia; ^11^Telethon Kids Institute, Perth, WA, Australia; ^12^Faculty of Medicine, The University of Queensland, Brisbane, QLD, Australia

**Keywords:** stillbirth, Aboriginal and Torres Strait Islanders, Indigenous, grief and loss, perinatal loss, antenatal care, maternal and infant health, Sorry Business

## Abstract

The stillbirth rate among Aboriginal and Torres Strait Islander women and communities in Australia is around double that of non-Indigenous women. While the development of effective prevention strategies during pregnancy and improving care following stillbirth for women and families in communities has become a national priority, there has been limited progress in stillbirth disparities. With community permission, this study aimed to gain a better understanding of community experiences, perceptions, and priorities around stillbirth. We undertook an Indigenous researcher-led, qualitative study, with community consultations guided by a cultural protection protocol and within an unstructured research framework. A total of 18 communities were consulted face-to-face through yarning interviews, focus groups and workshops. This included 54 community member and 159 health professional participants across remote, regional, and urban areas of Queensland, Western Australia, Victoria, South Australia, and Northern Territory. Thematic analysis of consultation data identified common themes across five focus/priority areas to address stillbirth: Stillbirth or Sorry Business Baby care needs to be family-centered; using Indigenous “ways of knowing, being, and doing” to ensure cultural safety; application of Birthing on Country principles to maternal and perinatal care; and yarning approaches to improve communication and learning or education. The results underscore the critical need to co-design evidence-based, culturally appropriate, and community-acceptable resources to help reduce existing disparities in stillbirth rates.

## Introduction

Stillbirth is a serious public health problem with far reaching psychosocial and financial burdens for families and communities (1). In Australia, there were 315,705 babies born in 2021, with ~2,278, or 76% of perinatal deaths due to stillbirth (2). This equates to *six* babies being stillborn every day (birth of a baby without signs of life after 20+ weeks or weighing 400+ grams (3) making stillbirth the most common form of perinatal/infant death across the general Australian population (4, 5). There have been minimal changes in stillbirth rates in the last three decades and persistent disparities exist across high income country settings.

The 2021 Australian Census reported that 812,728 people had identified as being of Aboriginal and/or Torres Strait Islander origin, representing 3.2% of the total Australian population of 25,422,788 people (6). The stillbirth rates among Aboriginal and Torres Strait Islander (herein respectfully referred to as Indigenous) communities is typically about twice that of the general population (12 compared with 7.2 per 1,000 births, respectively) (2, 5) and there has been limited progress in reducing this disparity (2, 5, 7, 8).

Furthermore, when an Indigenous baby is stillborn, appropriate care models that consider women's cultural needs may be ill-understood, and often suboptimal, resulting in a greater burden of grief on Indigenous communities (9, 10). Data on causes of stillbirth in Indigenous communities indicate that many stillbirths are preventable (9). This indicates a greater need for improvements in maternity care and raising community awareness on prevention and care during pregnancy (1, 3, 9). However, limited resources are available for health professionals to provide both culturally responsive information and care and respectfully consider traditional practices around stillbirth prevention (7). Prevention of stillbirth and pregnancy care requires systemic change in the healthcare environment, ensuring co-led stillbirth risk reduction campaigns with Indigenous community-controlled organizations and service providers (7). The Stillbirth Center for Research Excellence (CRE) is working to improve prevention and care for Indigenous people when such losses occur (11), along with organizations such as the Stillbirth Foundation Australia (12), Red Nose (13), and Still Aware (14) focusing on awareness, education, research, and advocacy. Prior to the introduction of the Safer Baby Bundle, and with the exception of some Still Aware resources (14), there were very few robust resources available on the prevention of stillbirth (15).

Following the Senate Enquiry into stillbirth research and education (4) the National Stillbirth Action and Implementation Plan was launched (16). The Plan aims to reduce stillbirth rates by 20% or more from 2020 to 2025 in Australia and to improve bereavement care through various ways including calls for a concerted national public awareness campaign (17). Furthermore, the Plan recognizes the significant equity gaps in stillbirth prevention and care, including those that exist between mainstream populations and Indigenous peoples and members of some migrant and refugee communities (5). The Safer Baby Bundle is the national stillbirth prevention initiative of the Plan. The Safer Baby Bundle focuses on five elements of maternity care practices where evident-practice gaps exist: smoking cessation support; improving detection and management of fetal growth restriction; improving awareness and management of women with decreased fetal movements; provision of maternal safe sleeping advice; and improving decision-making around timing of birth for women with risk factors (18). However, these resources were developed for stillbirth prevention for the general Australian population and are unlikely to be effective in Indigenous populations (10, 11).

The recent “Still Six Lives” national media campaign used digital marketing and social media to increase awareness of stillbirth and educate on the three modifiable behaviors during pregnancy to reduce the risk of stillbirth; its evaluation showing some evidence of effectiveness in increasing the proportion of Australian women who were aware of these three evidence-based preventive actions that reduce the risk of stillbirth (19). Pollock et al. (20) reported in their study there were limited levels of knowledge, attitudes, and perceptions of stillbirth in the general population, which warranted the need for future public health campaigns. Furthermore, the study highlighted the need for future co-designed research that “addresses the culture, values, needs and wants of a stillbirth public health campaign” with Indigenous peoples (20). Therefore, it is critical to frame stillbirth risk messages to ensure they are relevant in an Indigenous cultural context, are tailored to a family-centered approach and also to the realities of women's and families' lives (7). Actions to address equity gaps such as the recommendation for culturally appropriate models of care during the perinatal period (16) can be achieved through consultation and partnership in stillbirth prevention and care with Indigenous Australian communities and health services, including resource co-design and implementation. Therefore, understanding Indigenous people's experiences, perceptions, and priorities around stillbirth is key to implementing effective culturally safe prevention strategies in Australian maternity health services.

This paper aimed to identify needs around maternity care to prevent stillbirth with and for Indigenous Australian peoples. The second aim of the paper was to identify appropriate language for the term stillbirth with Indigenous communities.

## Methods

### Project foundation and values

This collaborative consultation process was led by dedicated, experienced Indigenous researchers, who have deep cultural connections with the lands and communities they come from. Indigenous research team members followed a strengths-based approach, amplifying the voices, experiences and needs of Indigenous women, families, and community members, harnessing and highlighting the strengths and resilience of Indigenous people. Researchers used yarning methods to provide a comfortable, safe space for community members to share stories, ask questions and feel heard. Indigenous researchers ensured Indigenous “ways of knowing, being, and doing” were honored (21). Relationship building with certain Aboriginal and Torres Strait Islander communities and Aboriginal Community-Controlled Health Organizations (ACCHOs) in South Australia, Far North Queensland, Victoria, New South Wales, and Western Australia occurred from 2019 to 2022 and explored community experiences, perceptions, and priorities in relation to stillbirth prevention and maternity care.

This paper reports on consultations with Indigenous communities around Australia, which formed the qualitative component (Phase 1) of a wider program of work—the cultural adaptation of the Safer Baby Bundle for Indigenous communities. Community consultations informed resource co-design approaches for an Indigenous-led cultural adaptation of the Safer Baby Bundle resources for community, and health professional educational resources, which will be reported in a future publication.

### Reflexivity of researchers

The Stillbirth CRE Indigenous Research Team is made up of Senior Advisor and Chair of the Stillbirth CRE Indigenous Advisory Group (IAG), Deanna Stuart-Butler (DSB), a descendant of the Arabana people of the “Pantu Parnda” (Lake Eyre) Region of South Australia. Deanna was leader of the Aboriginal Family Birthing Program in South Australia and conducted the consultations in Far North Queensland and South Australia. Carolyn Lewis (CL), a Yamatji Noongar woman and Aboriginal Research Fellow at Curtin University, led the consultations across Western Australia. Skye Stewart (SS), a Wergaia and Wemba Wemba midwife from Mallee Victoria, midwife and Research Coordinator led consultations in Victoria in discussion with Victorian Aboriginal Community Controlled Health Organization (VACCHO). Diana Jans (DJ), an Indigenous researcher with cultural connections to Far North Queensland undertook consultations in the Cape York region with DSB. Diana is a teacher, social worker, bereavement counselor and Narrative Therapist who has worked in human and child protective services and Apunipima Cape York Health Council in maternal, child and adolescent health since 2014. Senior research and clinical academics with extensive experience in midwifery, perinatal health (VF, PM, FB, SV) and Indigenous health (KMR, CS, SV) were integral in setting up and guiding the study. With expertise working in Indigenous health, SV worked closely with the team consulting in Queensland from the outset and played a major role getting the study underway, in consultations and qualitative analysis with the team. Early career researchers with experience in Indigenous health, perinatal health, and qualitative research (LM, RG, AB, LJ) were also part of the Stillbirth CRE Indigenous Research and authorship team. The Stillbirth CRE's National Indigenous Advisory Group, made up of Indigenous leaders in the maternity and infant health space, provided guidance and leadership from the outset of the community consultation work.

### Study design

The study was guided by Participatory Action Research principles (PAR) (22–24), and Indigenous-specific collaborative frameworks (23, 25, 26). Participants were interviewed via open individual and/or group discussions, usually as part of yarning circles—a culturally appropriate method of data collection. Sharing stories through yarning circles has been a way of learning for Indigenous people throughout time (21, 27). These stories often reflect the participant's lived experience, affirm identity, and allow sharing of each other's life and culture (26). While yarning, Indigenous people weave stories together, which enables the listener to make family, community, and Country connections (21). Yarning is a process which occurs in various forms, and includes social, research, and therapeutic yarns and can often lead from one into the other (27). In yarning, Indigenous people begin with a social yarn, discussing social connections, and establishing trust (27). Then, in a consultative or research yarn, the researcher and participant/s talk freely on an agreed subject without being limited by directive questions. Stories are often featured, which may provide a layered illustration of multiple issues that can be explored from various angles (28). It is a way of showing respect, building trust and often can promote healing when discussing a sensitive issue like stillbirth (26). Yarns conducted were also at times therapeutic yarns (27), allowing the opportunity for women and family members to talk about their loss.

Consultations and yarning were facilitated by up to four Aboriginal researchers (DSB, DJ, CL, SS), and at times one or two non-Aboriginal researchers (SV, AB) with community members and Indigenous and non-Indigenous health care professionals caring for Indigenous women and families. Prior to consultations a group yarning/interview discussion guide was developed to help guide conversations. Sample questions from the yarning/discussion guide included: *Do you think stillbirth is discussed with Aboriginal and Torres Strait Islander women during pregnancy? What is the best way to share information about stillbirth and provide support for Aboriginal and Torres Strait Islander women during pregnancy? What education/training is needed for clinicians? Are there current gaps in educational resources for community and health professionals? Is stillbirth an appropriate name for us to use in our communities?* Although care after stillbirth was not the focus of the study, it is not possible to talk about stillbirth prevention without also discussing the devastating consequences of stillbirth. Therefore, a question around care after stillbirth and/or the community experience of stillbirth was also included. The discussion guide focused the consultations on stillbirth risks and prevention, and opportunities on how resources should be developed for community needs.

### Context, collaborative consultation process and approach

The COVID−19 pandemic led to travel restrictions from 2020, which adversely impacted on the project timing and ability of the research team to visit many regional and remote communities. For this reason, a pragmatic approach was followed for the nationwide consultation. The CRE Indigenous Research Team decided to consult in targeted locations, that allowed for a national perspective ensuring meaningful consultations occurred at each site, while also prioritizing community safety during the pandemic. Face-to-face consultations with health professionals and community members were held in Far North Queensland (Cape York region), Western Australia, Victoria, New South Wales, and South Australia. The consultations started in 2019, and were finalized in 2022, including a delay of ~10 months due to COVID-19 pandemic disruptions (throughout early 2020—late 2021). During this period travel restrictions included limited travel between some states as borders closed, particularly Queensland and Western Australia, as well as the restriction of visitors into many Indigenous communities to stop virus spread in remote areas. Online platforms were used at times; however, face-to-face discussions were preferred by community and therefore prioritized, resuming in late 2021/22.

The pragmatic research consultation meant that data collection encompassed several different approaches, including collecting data through forums, workshops, focus groups, face-to-face and at times online yarns. Consultations in some regions involved face-to-face information sessions on stillbirth and the Safer Baby Bundle resources (18) followed by informal discussions on how to adapt stillbirth prevention information with Indigenous communities. The regions consulted were urban, regional, rural, and remote areas, to ensure the inclusion of a wide breadth of experiences and perspectives.

### Selection of participants

The choice of participant groups was based on existing relationships and networks between the Indigenous Research Team and ACCHOs and other health service organizations in South Australia, Victoria, Western Australia, and Queensland. The consultations focused on two participant groups: (1) Community members including Indigenous women of reproductive age (18 + years of age), men, and Elders; and (2) health care professionals, including maternity health professionals, and social and emotional wellbeing workers based in community health centers and clinics, program managers and policy makers, researchers working with Indigenous women in pregnancy and others who work with Indigenous communities. This included participants with lived experience of stillbirth. Staff information sessions at different locations were offered as both drop-in and open yarns, rather than structured interviews or focus groups. The two participant groups were interviewed/yarned separately, and some health care professionals who also identified as members of Indigenous communities provided feedback as both a community member and a health care professional, at the individual's discretion.

### Data collection

Consultations included group and individual interviews/yarns held at health services, community centers and when requested at community members' homes. Most consultations were audio recorded, transcribed verbatim and all were de-identified. A small number of consultations were not recorded, at the discretion of Indigenous Researchers conducting the yarn. This may have been due to the sensitive nature of the topic of stillbirth and may also have been due to location of yarn and the comfort level of participants to speak freely with a recording device present. However, extensive field notes were taken on the discussion, topics covered, and stories shared.

### Data analysis

Prior to coding, transcripts were checked for accuracy against the recording and de-identified. Analysis and interpretation were conducted collaboratively with the Stillbirth CRE's Indigenous Research Team using an iterative process. Indigenous Team members who collected the data, collaborated to refine the themes, and led the interpretation of the consultation data. Qualitative data from yarning circles and interviews were analyzed to identify themes using Framework Analysis (29). The Framework method offers a structured but adaptable approach to the thematic analysis of qualitative data (29). This method allows for the use of a pre-existing framework or set of key questions but is flexible enough to take account of new or differing concepts or issues that arise in the process of collecting data. To ensure conceptual consistency and interrater reliability at least two members of the research team conducted the analysis. The qualitative data analysis was conducted using NVivo 12 (QSR), and Word tables (Microsoft 365 Office). A summary of thematic findings and illustrative quotes and anecdotes are reported in Results.

## Results

The analysis in this paper reports on the qualitative data collected from group and individual interviews and yarns conducted face to face with 135 participants in Far North Queensland (FNQ), South Australia (SA), Victoria (VIC), and Western Australia (WA); these were specifically aligned with the stated aims of this paper (see Table 1 for participant characteristics). These interviews form part of a broader set of consultations which were held in 18 communities, with 54 community members and 159 health care providers (many of whom also identified as Indigenous community members), across remote, regional, and urban areas of Australia (see Supplementary Table 1 for an outline of specific consultation characteristics).

**Table 1:** Participant characteristics—roles and Indigenous status.

**Participants role**	**Female**	**Male**	**Total participants**	**Participants who identified as Indigenous**
**Far North Queensland**				
Health professionals^a^	25	4	29	22
Community members, incl. Elders	4	1	5	5
**Totals FNQ**	**29**	**5**	**34**	**27**
**South Australia**				
**Consultation 1**				
Health professionals^a^	-	-	-	
Community members, incl. Elders	24	-	24	20
**Total—Consultation 1**	**24**		**24**	**20**
**Consultation 2**				
Health professionals^a^	15	-	15	13
Community members, incl. Elders	2	-	2	2
**Total—Consultation 2**	**17**	**-**	**17**	**15**
**Totals SA**	**41**		**41**	**35**
**Western Australia**				
Health professionals^a^	33	**-**	33	30
Community members, incl. Elders	16	1	17	17
**Total WA**	**49**	**1**	**50**	**47**
**Victoria**				
Health professionals^a^	2	-	2	2
Community members, incl. Elders	4	4	8	8
**Total VIC**	**6**	**4**	**10**	**10**
**Overall totals**	**125**	**10**	**135**	**92**

a Midwives, Aboriginal Health Workers, managers, policy makers, researchers, and other health professionals.

Cell counts with < 5 were not tabulated to ensure confidentiality. Bold values indicate subtotals and total numbers of participants.

### Far North Queensland consultations

In total, 34 participants who ranged in age from 20 to 80 years took part in the consultations. Participants were predominantly female (85%); 55% identified as Aboriginal; 9% as Aboriginal and Torres Strait Islander or Torres Strait Islander; and 36% identified as non-Indigenous participants (from a variety of cultural backgrounds). Ten interviews were completed in this region, and all participants identified that even if they personally had not experienced stillbirth in their own life, they knew someone who had. Communities visited in the Cape York region included Weipa, Napranum, Mapoon, and Hopevale, with these sites chosen to provide a good representation of the community and the affiliation of the research team to Apunipima Cape York Health Council (the ACCHO for the Cape York region).

### South Australia consultations

There were two consultations held in SA with the first consultation facilitated by the Aboriginal Communities and Families Health Research Alliance (ACRA) in Adelaide (Tarndanya), with 24 attendees including service providers, program managers, policymakers, planners, and researchers working with Indigenous families during pregnancy. The focus of the sessions was on stillbirth research priority setting for Indigenous families, review of current practice, cultural safety, and stillbirth within Indigenous birthing programs. The second consultation was held in Adelaide with 17 Aboriginal Maternal and Infant Care (AMIC) practitioners, health care providers and a community member. The session discussed how stillbirth prevention, care and bereavement care fit into the AMIC principles that guides their work; to ensure optimal care for Indigenous women and families in South Australia.

### West Australia consultations

Four yarning circles were undertaken with community members and health services staff of rural, remote, and urban areas of Western Australia to elicit knowledge and experiences of stillbirth, risks, prevention, and care. Two of the four yarning circles held were two-way knowledge exchange workshops led by Elders and the Indigenous Researcher and conducted with Indigenous and non-Indigenous health professionals, community members, and families. Consultations in WA were held in the following locations: urban—Derbarl Yerrigan Health Service, East Perth (Booroolo); regional—South-West Aboriginal Medical Services (SWAMS), Bunbury; rural/regional—Geraldton Regional Aboriginal Medical Service (GRAMS), Geraldton and Bega Garnbirringu Health Service (BEGA), Kalgoorlie.

### Victoria consultations

Five consultations with eight community members and two health services staff were held in rural, regional, and urban areas of Victoria to elicit their awareness, knowledge and experiences of stillbirth, risks, prevention, care during and after stillbirth. Consultations were held in various locations including urban—Melbourne (Naarm); rural/regional—Warnambool (Gunditjmara); Swan Hill (Wamba Wamba); Sea Lake (Wergaia); online consultation—with participants from bordering regional/rural area of NSW and Victoria.

## Qualitative results

### “Sorry Business Babies”—language for stillbirth

A key aim of the study included identifying a culturally appropriate term for stillbirth. “Sorry Business” is a term used by Indigenous Australians to encompass the time of traditional rites and customs related to death; it is a mourning period when a family or community member dies and describes all responsibilities that follow in accordance with traditional lore and custom, such as grief, loss and funerals (30, 31). Stillbirth is referred throughout this work as “Sorry Business Baby” or “Sorry Business Bubba.” In other regions they have been referred to as “Star Babies.” We started with the concept of Sorry Business and refined the use of this term after early consultations. “Sorry Business Bubba” was the term used by the Stillbirth CRE Indigenous Research team when referring to stillbirth, or when a baby passes away after 20 weeks of pregnancy or during birth.

*When a woman and family are going through Sorry Business after having a stillborn baby, they suffer tremendous grief and loss. It is also a difficult time for their health carers. Stillbirth is a sensitive issue in Aboriginal and Torres Strait Islander communities—there is little open discussion*. (Deanna Stuart-Butler, Stillbirth CRE Indigenous Research Team Senior Advisor)

Based on consultation findings and early guidance from the Indigenous Advisory Group, the term was used by CRE Indigenous researchers to enable more open yarning around the taboo topic of stillbirth. The term was also used in the cultural adaptation of the Safer Baby Bundle project work.

*Know that after stillbirth, Aboriginal families are in Sorry Business which is a cultural rite and responsibility. It means that we grieve collectively and there may be practices we do at this time to honor the bub who has passed. It might mean that we need more time when talking to us about next steps, like autopsy and other stillbirth investigations. It will be hard for family to make quick decisions. Sorry Business goes for days, weeks, or months and it doesn't matter if it is a bubba or an Elder, the Sorry Business and life of that person is equal*. (Aboriginal midwife and woman who experienced loss, VIC)

Awareness of the sacred nature of Sorry Business was identified as culturally appropriate practices that helped families after stillbirth, and were an important facilitator during consultations.

*Culture is the protective factor for Aboriginal people. It is what keeps us well and safe. The death of anyone in Aboriginal community means there is a time of Sorry Business, which is a time of cultural rite and responsibility*. (Skye Stewart, Stillbirth CRE Indigenous Research Officer and Midwife)

### Thematic findings—five priority areas

**Figure 1:**
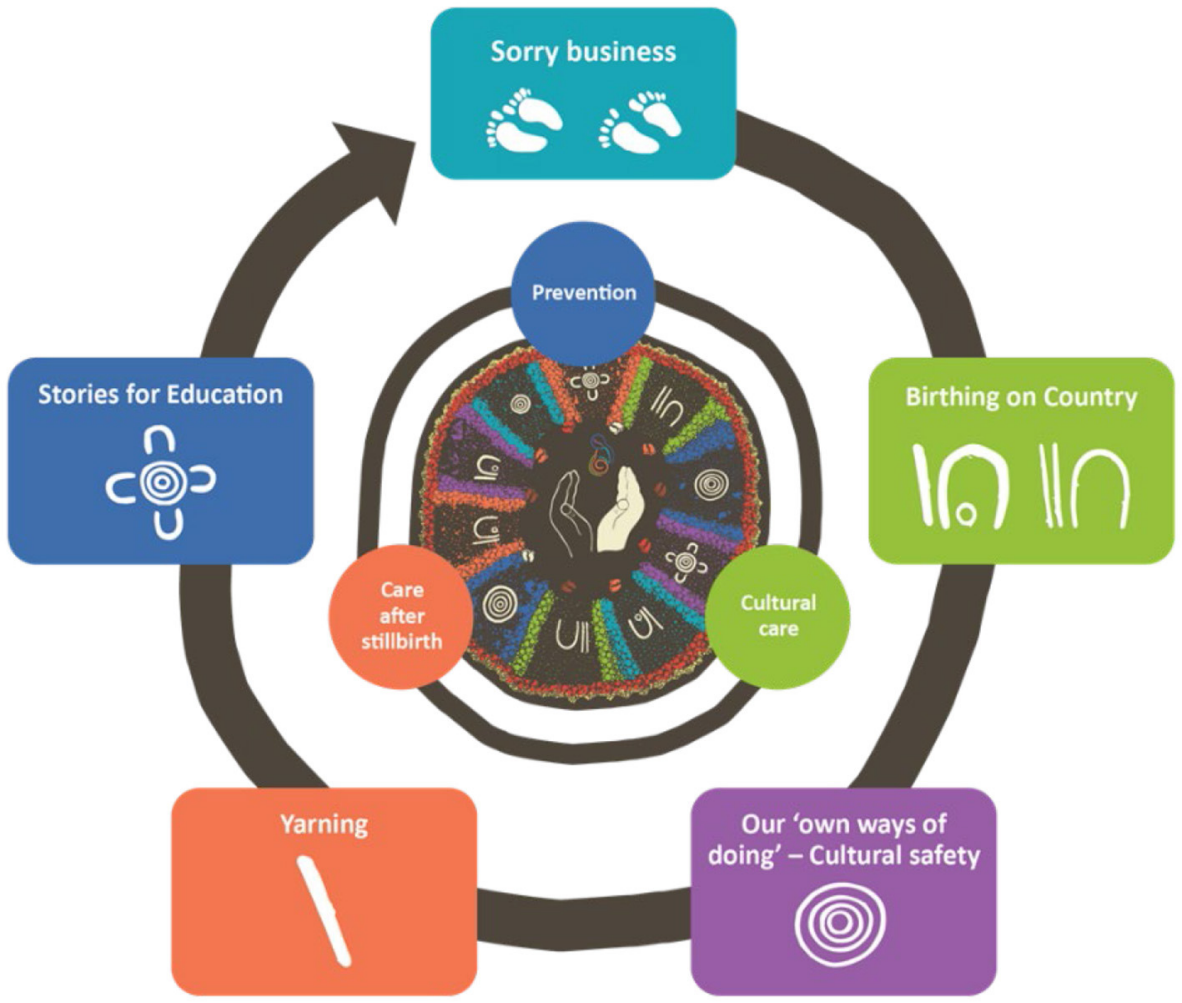
Five focus areas based on thematic analysis of consultations.

Overall, thematic analysis identified five key themes to address improvements in stillbirth prevention and bereavement care. These included: (a) Stillbirth or Sorry Business Baby care needs to be family-centered, (b) Using Indigenous “ways of knowing, being and doing” to ensure cultural safety, (c) Application of Birthing on Country principles to perinatal care, (d) Yarning approaches to improve communication, and (e) Learning and education through stories (see Figure 1). The five priority themes and illustrative quotes from consultations across the four regions are outlined below.

#### Stillbirth or Sorry Business Baby care needs to be family-centered

From these consultations it was clear that family-centered care was paramount during a time of grief and loss when a baby is stillborn. Participants stated that grief and loss services were needed across urban, rural, and remote areas, recognizing that a focus on more than just “mum and dad” was required. Health care workers were needed to empower the whole family, including parents and other family members involved in the decision-making process for the baby:

*Lots of different things can happen and for [a] family to have the shock of their baby dying and then have to go through all these other things..*. “*I've got all these doctors and people wanting me and yet I've got all my family, who are wanting me to tell them what happened and how come?*”…* Lots of pressures. Having that [health service] support, is a really good idea*. (Aboriginal health care provider, Cape York FNQ)

*Definitely [there is] a lack of support service such a grief and loss services for the mother and other family members to access*. (Aboriginal midwife, WA)

Participants identified it was important that families feel enabled to make their own decisions for their stillborn baby, to avoid feeling scared and shamed, and to be able to ask for what they need—culturally, physically, and emotionally.

*It feels like Aboriginal community have some shame job around... talking about it. At the same time, it feels like health providers are awkward or don't want to start the conversation in case there are questions they don't know how to answer. What is required for families is for health providers to be confident and kind in their conversations, as it needs to be talked about*. (Bereavement worker, VIC)

Family-centered care encompasses both the mother and father and generally the extended family as well. Culturally, even though birth is Women's Business,^1^ participants outlined that there was a place for men in Sorry Business. This included how the health care team responded to the father or other male relatives. Participants indicated that men had responsibilities, not only in carrying their grief, but in supporting their partner. It is however important to note, that we recruited and only consulted with a small number of men, with the following reported by a male participant:

*When things are tough, we have to stay strong and if that means there is a stillbirth, we need to support our women, as well as somehow keep carrying on, even in our own grief. Sometimes we have our own traumas, and we are treated badly by the hospital staff, it makes us shame job. We don't need to carry shame; we are doing our best and we need support too*. (Aboriginal father, VIC)

#### Using Indigenous “ways of knowing, being, and doing” to ensure cultural safety

Embedding Indigenous “ways of knowing, being, and doing” to ensure a culturally safe and responsive practice was identified as a key theme in all locations. Indigenous “ways of knowing, being, and doing” encompass the following principles as expressed by Martin and Mirraboopa (21):

“*Recognition of our worldviews, our knowledges and our realities as distinctive and vital to our existence and survival; Honoring our social mores as essential processes through which we live, learn and situate ourselves as Aboriginal people in our own lands and when in the lands of other Aboriginal people; Emphasis of social, historical and political which shape our experiences, lives, positions and futures*; *Privileging the voices, experiences and lives of Aboriginal people and Aboriginal lands*.”

This was highlighted in our study as being especially pertinent in relation to adverse situations during pregnancy and birthing healthcare experiences. Participants identified a lack of cultural safety that included: disempowerment; health professionals not always communicating around the time of stillbirth, which was essential to ensuring women and their families' understanding; lack of trust in people and the health system resulting in less confidence to engage. Racism was experienced by Indigenous people in general health care settings, which significantly impacted on cultural safety. Application of culturally safe practices in regional health centers was questioned, including health care staff's understanding of community customs; willingness to consult the family about the cultural and other arrangements appropriate for them.

*We don't shy away from hard yarns but if we feel unsafe by systems or people around us, then we shut down. It is a coping mechanism. We have been so hurt in the past and we protect ourselves from harm. If the health providers talk to the Aboriginal community in a way that shows cultural integrity and safety in their practice, then it gives that room for us to feel alright to open up. Aboriginal people want to receive health care. We just want that health care to be safe for us*. (Community member/parent, VIC)

Discussions related to this theme also identified that organizational and individual responsibility to deliver culturally safe care is of paramount importance; care needs to be considered, acknowledging of individual differences; professionals need to engage in cultural humility and reflective practice to ultimately deliver culturally responsive and responsible care that women, families, and communities deserve. Aboriginal maternal and infant health care staff and Aboriginal health worker participants identified several areas of need:

*Aboriginal ways of knowing, being and doing surpass anything else and need to be at the center of the care and the decision-making process*. (Bereavement worker, VIC)

*I know with our mob [health professionals should] ask because sometimes those women [sic bereaved mothers*] *are never given the privilege of being asked if they want to keep the placenta. And umbilical cord…. You've got something else of the baby…now we seem to see this resurgence of wanting to go home on country, wanting to know where their birth tree is or cave or you know? So, my thing is, ask*. (Aboriginal and Torres Strait Islander Health Worker, female Elder, Cape York, FNQ)

Embedding Indigenous “ways of knowing, being and doing” has been recognized as a key feature in culturally-safe maternal and infant health programs and services run through ACCHOs, as they foster cultural connections for women and families (32–35). Aboriginal community-controlled health organizations aim to deliver services in a way that engenders trust and are more acceptable to local communities.

*As an organization [ACCHO], you know, we are about cultural safety and that honesty and an open approach. Otherwise, people will not trust us. If they don't trust us, then we may as well just stop doing what we do*. (Non-Indigenous doctor, male, Cape York, FNQ)

#### Birthing on Country principles for perinatal care

Birthing on Country is a metaphor for the best start in life for First Nations families (36). The term recognizes that when women give birth in Australia, they are doing so on the sovereign lands of the First Peoples of Australia who have never ceded ownership of their land, seas, and sky (36). Birthing on Country principles encompassed the delivery of holistic, continuity of care models for women and families, and where possible, for women to have a Cultural Primary Carer during pregnancy and beyond.

“*Aboriginal led antenatal care [AHW/AMICs] at all appointments and culturally appropriate models of care, including continuity of care, which women trust and feel comfortable with are important.”* [Aboriginal Maternal and Infant Care (AMIC) Worker, SA]

There is growing awareness and respect of the importance of Birthing on Country for Indigenous women, with a few initiatives now starting in maternity care in Australia (32, 35, 37). Birthing on Country not only refers to redressing the negative impact of colonization and returning childbirth services to Indigenous communities and control; it also encompasses services which are characterized as “community-based and governed; allow for incorporation of traditional practice; involve a connection with land and country; incorporate a holistic definition of health; value Indigenous and non-Indigenous ways of knowing and learning, risk assessment and service delivery; are culturally competent; and developed by, or with, Indigenous people” (36). As stated by a maternal and infant care worker:

“*Supporting women to birth on Country or birth within community—Birthing programs are a positive step and should be rolled-out wider.”* (AMIC worker, SA)

However, some participants stated that there were and continue to be challenges applying Birthing on Country principles reported, such as the management and loss of continuity of carer, with many women birthing at the hospital, which is a separate service and therefore without the ACCHO doctors and midwives, that provided their antenatal care at their local ACCHO. This is especially the case for women having to travel from remote and rural locations to birth in larger regional and urban maternity hospitals for delivery and/or manage later pregnancy complications. The practice of relocating women away from home for birthing presented additional challenges.

*I think it's a combination of history and culture and a mistrust of organizations, and the fact that people have been let down possibly in the past…I think it's a loss of cultural norms for people who would have birthed on Country, and Elders in community, female Elders in community would have managed a lot of those processes. And then we've had the whole swathe of the western lifestyle negative influences coming in, you know, which have just complicated people's pregnancies and made it so much more difficult*. (Non-Indigenous doctor, male)

#### Yarning approaches to improve communication

“Yarning” methods are recognized as a culturally appropriate way of collecting information or data with Indigenous women and their families (27). Participants reported that when Indigenous families felt safe and cared for, they might be more likely to share. Therefore, health care practitioners were advised to provide space and opportunities for amplifying the voices of Indigenous peoples in their care. It was important that families knew that they were in control and able to make their own decisions. Using “strengths-based language or approaches” refers to those approaches that have their foundations in Indigenous ways of knowing, being and doing, and “view strengths not as the possessions of individuals, but as the structure and quality of the social relationships, collective practices and identities that are present in Indigenous communities (38). Strengths-based approaches should therefore be used in yarning with community about health. This was however identified by health professionals as an area in need of improvement.”

*Stillbirth is sad, how do you communicate effectively?* (non-Indigenous midwife, WA)

*First thing [the non-Indigenous health care provider] said off the top of their head to them “you've got uncontrolled diabetes so you're at risk of stillbirth” and that woman is just sitting there, and you sort of sit there too and think ‘Did [they] just really say that out loud?' and there was no care, it was just like [it's a] pre written record*. (Indigenous AMIC practitioner, SA)

Indigenous families highlighted that medical conversations, particularly around investigations after stillbirth, needed to happen however it was reinforced that it was important that the care from health professionals was culturally safe throughout the entire pregnancy. Aboriginal Health Workers (AHWs) were crucial for having a yarn with families and must be available at hospital at a time of Sorry Business. They can explain medical terminology, speak up and ask questions on behalf of the parents.

*AHW/AMIC continuity of care to support women to understand and translate medical information [is needed]*.

*Education for non-Indigenous health workers to have conversations around Aboriginal culture and practices [is needed]*. (AMIC worker, SA)

Stillbirth/Sorry Business Babies is a difficult topic and is generally understood to be a taboo subject to talk about in Indigenous communities. Therefore, a two-way exchange such as through yarning approaches needs to take place between health professionals and bereaved families. Open clear communication from the health care practitioner, in a way that is safe and respectful and holds cultural integrity is paramount:

*Information that is new to you is hard to digest when you are talking about your precious baby that died. Talk calmly and don't rush the family if you are the health care practitioner caring for them. Just allow quiet space and don't expect too many words or for us to give the answers all at one time*. (Bereavement worker, female, VIC)

*If the doc needs to refer… [and] there's this issue, I say “Can you change it to just you need someone to yarn to.”* (Aboriginal and Torres Strait Islander Health Worker, female Elder, Cape York, FNQ)

#### Learning or education through stories

Indigenous cultural practices which have positive effects on children and communities include kinship relations, oral traditions that are often centered around stories, traditional knowledge systems, a collective community focus with respect for Elders' contributions, and spirituality (27, 39, 40). Ideas were highlighted such as the need for more Indigenous health professionals who have lived experience and knowledge of history and culture, who can share this through their stories for strengthening education. Additionally, stories could be used by non-Indigenous health professionals when explaining sensitive topics or medical information.

*Education for clinicians—around complex trauma, how historical trauma, trauma associated with coming into a hospital, etc. may compound distress*.

*Education for women/families on causes, and that often a stillbirth is unexplained, and no one's fault, is important to help women and families through the guilt after a stillbirth*. (AMIC worker, SA)

## Discussion

We have reported on the processes undertaken by the research team that ensured a thorough and robust consultation around a sensitive topic to ensure representation from various Indigenous communities in most Australian States and Territories. Like other researchers and community leaders before them, this team led by Indigenous researchers, acknowledged that Indigenous women are not a homogenous group, rather they are characterized by differences in culture and traditions, languages, socio-economic circumstances, places of residence, educational levels, and employment status (40, 41). Despite the challenges imposed by the COVID-19 pandemic this team undertook a pragmatic approach and gained significant insights into this unmet need. The work highlighted the need for a culturally appropriate term for stillbirth and the yarning approach around the topic elicited communities' perspectives on a culturally appropriate term. Our findings have underscored that there are minimal culturally specific resources available, and limited understanding about responsive, culturally safe maternity and maternal and infant health services, particularly related to stillbirth for Indigenous people. The priority focus areas identified through this thematic analysis will consolidate approaches needed to address stillbirth in Indigenous communities. This which will include the next phases of development of appropriate stillbirth prevention resources based on the Safer Baby Bundle for Indigenous people.

Conducting respectful, consultative, and meaningful health research with Indigenous communities has been especially important when consulting around sensitive and less often spoken about topics such as stillbirth. Birthing on Country and continuity of midwifery care are key recommendations for community-based solutions to Closing the Gap in birthing and health inequalities that exist for Indigenous peoples (32, 35, 37). Reducing the stillbirth rate and improving the general health and wellbeing of Indigenous women is a key principle of Birthing on Country approaches (4, 32). Our work in this space, as well as the work of others in other health areas, highlights the importance of understanding these experiences from the perspective of the Indigenous communities to ensure culturally appropriate conversations about prevention strategies. Indigenous pregnant women who live in rural or remote areas may need to be transferred to the nearest regional or urban hospital for delivery, away from their family and community. This results in women being away from their connection to their cultural lands and Country, as well as being transported while delivering a Sorry Business baby into an unfamiliar health care team and system, that may not understand or recognize the importance of cultural practices during this time of grief and loss. For many Indigenous women and their families, delays, or prevention from undertaking these practices fundamentally escalates the stress of this event, and gentle supportive healthcare could help to alleviate this.

Our trustful, respectful, and thorough consultation process allowed for identification of contentious issues, gaps in awareness and education, and research priorities. Across all topics discussed during the national consultation undertaken, lack of continuity of care was identified as the main barrier in effective stillbirth prevention, investigation of stillbirth causes, and care for families following stillbirth. The importance of ensuring midwives and service providers have the education to provide continuous care for a family was noted as the main goal of caring for Indigenous families during the perinatal period. Recommendations focused on education and training for health care workers to support and empower pregnant women and their families. The yarning sessions undertaken also served to explore the feasibility and acceptability of education, training, and the development of culturally appropriate resources aimed to support Indigenous communities. Guidance for those undertaking future research based on our study findings includes the critical need to co-design evidence-based, culturally appropriate, and community-acceptable resources to help reduce existing disparities in stillbirth rates in Indigenous communities.

### Lessons learned in conducting this research

Insights from this work can be translated to other settings include the need for: Indigenous leadership in the research process; flexibility and realistic time frames when working with Indigenous communities to support cultural protocols and priorities. During this project the COVID-19 pandemic period provided an extreme case where the team needed to manage several challenging conditions to support health and safety of Indigenous communities. Further learning's included understanding of diversity of Indigenous communities, understanding the distance and requirements of travel out to communities around the country. In our efforts to ensure face-to-face communications it became the project responsibility to undertake the travel to remote communities and gave the team additional insights into the challenges communities face to manage when there is a need for birthing elsewhere. A recommendation for researchers to attend more community yarning sessions to meet with women's groups, men's groups and visit more rural and remote communities was suggested. Inclusion of more men in the research process would have been of benefit, and having an Indigenous male to lead this, as well as having visible male representation in the development of future resources was also recommended.

### Challenges

Although we have captured a series of consultations during this project, we did encounter a few challenges, the biggest being the COVID-19 pandemic and ensuing travel/access restrictions. Indigenous women, families, and communities already faced disadvantages in inequitable access to health care, and the COVID-19 pandemic exacerbated this in many instances. Although not relevant to our study, the pandemic further decreased access to health care in remote regions due to limitations on the ability to enter specific regions across the country and to visit communities during lockdown periods, including for researchers.

A particular challenge faced by the research team was the occasional pressures placed by the funder's deliverable timelines, and their limited understanding of culturally appropriate consultation processes. As is often the case, funding bodies have an expectation that research projects will deliver to a timeline of deliverables and apply significant pressure for this to occur. To enhance this understanding, a workshop was conducted by Stillbirth CRE Indigenous researchers and funding body representatives in September 2022. At times, travel restrictions from COVID-19 pandemic period changed extremely quickly and meant cancellations and not being able to reach more communities as planned. Unfortunately, there was little sympathy for this and continued to add to the burden of stress that this work entailed. The research team's commitment to follow Indigenous ways of knowing, being, and doing became a challenge in the circumstances, as it meant that significant education had to be provided to the funding body to ensure a culturally appropriate process continued.

During the pandemic period, there were occasional difficulties in securing health service staff to attend consultations due to prioritizing health care delivery for COVID-19 care. Similarly, it was at times challenging to have Indigenous community members participate due to the sensitive nature of the topics to be discussed. Despite these challenges, we were able to capture several viewpoints of women and family members with lived experience or awareness of others' experience of Sorry Business Babies, and health professionals who cared for women during pregnancy. To counteract the potential distress caused by the topic stillbirth, we included a follow up of women interviewed as needed and offered support by Social and Emotional Wellbeing Teams and/or by the Employee Assistance Program.

### Opportunities

To overcome these challenges due to the COVID 19 restrictions we engaged with some community participants virtually. However, many Indigenous communities' encountered difficulties in access to equipment and internet connection. Further, there was some reluctance to use it, as for many people it was their first experience of these virtual meeting spaces. Virtual consultations were not as effective as face to face as it challenged the idea of a natural and supportive environment and restricted the flow of Indigenous ways of yarning especially when disclosing something as personal as a Sorry Business Baby.

From our consultations we recognized a gap in information available for Indigenous women who continue to have disproportionate burden of stillbirth in Australia and have needs requiring careful consideration in care after stillbirth. This project set out to explore experiences of stillbirth from the perspectives of Indigenous women and their communities, through discussions with community members and health professionals of Far North Queensland, South Australia, Victoria, and Western Australia. The Stillbirth CRE Indigenous Research Team worked with community level permission and direction, using yarning methods in data collection, and ensured data sovereignty through community ownership, and community benefit.

## Conclusion

Our findings outlined the five key areas of focus to ensure stillbirth prevention and care of Indigenous peoples is culturally safe and responsive. Our study included a detailed consideration of Indigenous peoples' experiences of stillbirth and awareness of stillbirth risks, which to our knowledge, was the first time this has been undertaken with Australian Indigenous populations at scale. The findings provide a foundation of understanding for the development of culturally appropriate awareness and prevention resources and will support efforts to adapt or create existing resources. Consultation findings helped to inform and guide the co-design of culturally responsive resources for pregnant women to reduce stillbirth rates, and support families after the loss of their baby, and are an important step in addressing stillbirth prevention and care (42). Areas of future development include continued work to ensure Indigenous parents and community members have their voices, experiences and needs around Sorry Business babies heard at all levels within the policy-setting process through enabling conversations, and the development and dissemination of culturally appropriate resources, including in bereavement care. This research aims to contribute to the evidence-base in working toward optimal care of Indigenous families.

## Data availability statement

The raw data supporting the conclusions of this article will be made available by the authors, without undue reservation.

## Ethics statement

This study was approved by the following Aboriginal-specific human research ethics committees in South Australia, and Western Australia, and included one with an Aboriginal review committee in Queensland: South Australian Health and Medical Research Institute, and South Australian Aboriginal Health Accord (^***^) (South Australia); Far North Queensland Human Research Ethics Committee (^***^); Western Australian Aboriginal Health Ethics Committee (HREC1073); Mater Misericordiae Ltd Human Research Ethics Committee (HREC/MML/^****^) and ratified by one university Human Research Ethics Committees (^***^). Due to the sensitive nature of this topic, and to minimize harm during interviews, a risk and safety strategy for community members and staff was put into place, guided by social and emotional wellbeing counselors of participating health services.

## Author contributions

LM: Data curation, Formal analysis, Methodology, Project administration, Software, Writing—original draft, Writing—review & editing. CL: Data curation, Formal analysis, Investigation, Methodology, Validation, Writing—original draft, Writing—review & editing. SS: Data curation, Formal analysis, Investigation, Methodology, Validation, Writing—original draft, Writing—review & editing. DJ: Data curation, Formal analysis, Investigation, Validation, Writing—original draft, Writing—review & editing. RG: Data curation, Formal analysis, Methodology, Project administration, Software, Supervision, Writing—original draft, Writing—review & editing. LJ: Data curation, Formal analysis, Project administration, Software, Writing—original draft, Writing—review & editing. AB: Data curation, Formal analysis, Investigation, Software, Validation, Writing—original draft, Writing—review & editing. PM: Conceptualization, Data curation, Formal analysis, Methodology, Resources, Supervision, Validation, Visualization, Writing—original draft, Writing—review & editing. SV: Conceptualization, Data curation, Formal analysis, Investigation, Methodology, Supervision, Validation, Visualization, Writing—original draft, Writing—review & editing. FB: Conceptualization, Data curation, Formal analysis, Methodology, Validation, Visualization, Writing—original draft, Writing—review & editing. CS: Data curation, Formal analysis, Methodology, Resources, Supervision, Writing—original draft, Writing—review & editing. VF: Conceptualization, Data curation, Formal analysis, Funding acquisition, Investigation, Methodology, Resources, Supervision, Validation, Visualization, Writing—original draft, Writing—review & editing. DS-B: Conceptualization, Data curation, Formal analysis, Investigation, Methodology, Supervision, Validation, Visualization, Writing—original draft, Writing—review & editing. KR: Data curation, Formal analysis, Methodology, Resources, Supervision, Visualization, Writing—original draft, Writing—review & editing.

## Footnotes

^1^Women's Business refers to the practices, ceremonies, and Lore that are specific and sacred to Aboriginal and Torres Strait Islander women. Women's Business is performed separately from Men's Business and can relate to health, upbringing, ceremonies, and maintenance of culturally significant places and sacred sites; it is also a way of reconnecting with the ancient wisdom, tools, and culture to pass down the next generation of young women [Source: Cultural Ways—Learn—Australian Indigenous HealthInfoNet (ecu.edu.au)].
