# Supplementary material for: Looking after bubba for all our mob: Aboriginal and Torres Strait Islander community experiences and perceptions of stillbirth
Source: Front Public Health. 2024 Apr 16;12:1385125. doi: 10.3389/fpubh.2024.1385125 (PMC11059953; doi:10.3389/fpubh.2024.1385125)
Supplement: Supplementary file 1 [file Table_1.pdf]

Supplementary Table

Summary of consultations; format, location, and attendees

Consultation	Consultation Format	Location	Attendees	Date
**NATIONAL**				
**Virtual consultation**	Online	Online consultation	9 Aboriginal health care professionals	September 2020
**SOUTH AUSTRALIA**				
**Consultation one**	Face-to-face	Aboriginal Health Council, Adelaide South Australia	24 Aboriginal and Torres Strait Islander community members and Elders	February 2021
**Consultation two**	Face-to-face	SA State Forum with Aboriginal Health professionals from Urban: Adelaide<br>Regional:<br>Gawler, Murray Bridge and Port Augusta<br>Remote: Ceduna	17 Aboriginal Maternal and Infant Care (AMIC) practitioners	June 2022
**NORTHERN TERRITORY**				
**Consultation**	Hybrid (Face-to-face and online)	Northern Territory Safer Baby Bundle Launch held at the Royal Darwin Hospital	69 Health care professionals providing care to Aboriginal and Torres Strait Islander families during pregnancy	May 2022
**QUEENSLAND**				
**Focused consultations (10 in total)**	Face-to-face multiple consultations; (telephone)	Health services and community members' home/s	34 people who live and/or work in Cape York participated in individual and group interviews:<br>• 28 health professionals with diverse roles: Aboriginal and Torres Strait Islander Health Workers; and 1 Aboriginal and 7 non-Indigenous midwives working in the Maternal and Child Health Program. Other primary health care staff from the general and Social and Emotional Wellbeing programs.<br>• 5 Senior Aboriginal and Torres Strait Islander community members. One family invited the interviewers to their home, for a multigenerational discussion involving both females and males.<br>• 1 phone interview conducted with a senior member of Apunipima Health Service.	December 2020, March, and June 2021
**WESTERN AUSTRALIA**				

Consultation	Consultation Format	Location	Attendees	Date
**Focused consultations (23 in total)**	Face-to-face<br><br>Phone call and video conference<br><br>Workshop/ presentation	Health and community services	50 people participated in individual and group interviews with who live and/or work in rural, regional, and urban Western Australia<br><br>• 31 health professionals with diverse roles: 18 Aboriginal Health Workers and Hospital Liaison Officers; 1 Aboriginal and 3 non-Aboriginal midwives.<br>• 2 other staff: 1x Regional Aboriginal Health staff and 1x community organisation staff.<br>• 17 community members who were not health professionals; some were senior members of the community.	February - April, and Aug 2022
**VICTORIA**				
**Focused consultations (5 in total)**	Face-to-face and Zoom teleconference	Health and community services	10 people participated in individual and group yarns who live and/or work in rural and urban Victoria and regional NSW.<br><br>• 2 health professionals: Aboriginal Hospital Liaison Officer and Nurse.<br>• 8 Aboriginal and Torres Strait Islander community members who were not health professionals; some were senior members of the community, and some had lived experience of stillbirth.	April - June 2022
